# Treatment‐naïve people living with HIV aged 50 years or older in Beijing, China, 2010–2020: joinpoint regression model analysis of surveillance data

**DOI:** 10.1002/jia2.26193

**Published:** 2023-12-06

**Authors:** Duoduo Wang, Mengge Zhou, Peicheng Wang, Jinjuan Zhang, Yuanqi Mi, Feng Cheng, Jufen Liu

**Affiliations:** ^1^ Department of Rheumatology and Clinical Immunology Peking Union Medical College Hospital Chinese Academy of Medical Sciences Beijing China; ^2^ Institute of Reproductive and Child Health/National Health Commission Key Laboratory of Reproductive Health Peking University Beijing China; ^3^ Department of Epidemiology and Biostatistics School of Public Health Peking University Beijing China; ^4^ Department of Epidemiology and Biostatistics Institute of Basic Medical Sciences, Chinese Academy of Medical Sciences School of Basic Medicine Peking Union Medical College Beijing China; ^5^ Vanke School of Public Health Tsinghua University Beijing China; ^6^ Department of Epidemiology Bloomberg School of Public Health Johns Hopkins University Baltimore Maryland USA

**Keywords:** China, HIV, epidemiology, antiretroviral therapy, comorbidity, risk factors

## Abstract

**Introduction:**

As they age, people living with HIV (PLWH) must face new challenges, such as accelerated ageing and higher rates of comorbidities. This study described the characteristics of HIV acquisition among treatment‐naïve PLWH aged ≥50 years and <50 years in Beijing from 2010 to 2020, exploring associated risk factors for comorbidities.

**Methods:**

In this cross‐sectional study, differences in HIV‐related and non‐HIV‐related characteristics were compared using the *t*‐test, Mann−Whitney *U* test and chi‐square test. Temporal trend data were analysed via joinpoint regression. A multivariate logistic regression model was conducted to analyse the associated factors with PLWH having one or more comorbidities.

**Results:**

The proportion of PLWH aged ≥50 years has significantly increased since 2013, with a corresponding increase in homosexual transmission in this age group over the past decade. The proportion of individuals with CD4 counts <200 cells/μl significantly decreased from 2010 to 2013 among PLWH aged ≥50 years and from 2010 to 2014 among those aged <50 years. Delayed initiation of antiretroviral therapy (ART) improved for both age groups over the course of the decade, especially from 2014 to 2020. Compared to PLWH aged <50 years, those aged ≥50 years had a higher proportion of CD4 counts <200 cells/μl, higher levels of plasma HIV RNA load and a higher prevalence of non‐HIV‐related risk factors. Multivariate analysis revealed that PLWH aged ≥50, male, not single, transmission through heterosexual contact or drug injection, WHO Stage IV, coinfection with hepatitis B virus/hepatitis C virus and CD4 counts <200 cells/μl at the initiation of ART were associated with higher risk of the presence of an HIV comorbidity.

**Conclusions:**

Due to the persistent burden of HIV‐related characteristics or symptoms and the increasing prevalence of coexisting comorbidities among treatment‐naïve PLWH aged ≥50 years, physicians should provide the highest‐quality screening, prevention, treatment and management of coexisting comorbidities, adopting a multidisciplinary approach.

## 1 INTRODUCTION

Antiretroviral therapy (ART) has transformed human immunodeficiency virus (HIV) from a uniformly fatal to a chronic condition [1]. However, people living with HIV (PLWH) still have shorter life expectancies and greater comorbidities than the general population, particularly in the earlier stages. In 2019, globally, there were 8.1 million older PLWH aged ≥50 years, 22% of the total PLWH, and there were 180,000 newly diagnosed cases in this age group in 2019, accounting for 9.5% of the global incidence [2]. In China, PLWH aged ≥50 years accounted for 42.4% of cases in 2019, with the proportion of newly diagnosed PLWH aged ≥ 50 years reaching 28%, demonstrating a continuous upwards trend and well above the global average [2].

Although ART has significantly decreased HIV‐associated morbidity and mortality [3], AIDS‐related opportunistic illnesses (AOIs) remain common at presentation or during follow‐up and are a leading cause of hospitalization and mortality among PLWH [4]. In older patients, however, CD4 T lymphocyte levels recover more slowly, even with a good response to ART [5, 6]. This is associated with an increased risk for severe disease and mortality [7].

Older PLWH face other challenges as well, such as accelerated ageing and higher comorbidity rates [8]. A previous study reported that 83% of older PLWH had at least one comorbidity, and more than two‐thirds had two or more [9]. Managing comorbidities in older PLWH is challenging due to system barriers, unavailable HIV‐specific recommendations, epidemiological differences in some comorbidities and frequent interactions between ARTs and other drugs [1, 8]. In addition, stigma is a main driver of the HIV epidemic and is a significant barrier to the HIV care cascade. Older PLWH may experience both HIV‐related and age‐related stigma resulting in social isolation and lack of social support [10].

This study compared HIV‐related characteristics and comorbidities between ART‐naïve PLWH aged ≥50 years and <50 years to explore the associated factors with one or more comorbidities based on 11‐year surveillance data from Beijing.

## 2 METHODS

### 2.1 Study design and population

This was a cross‐sectional study, conducted using data collected from a surveillance system that tracks clinical information on PLWH receiving ART and long‐term follow‐up care at the STD/AIDS Prevention and Treatment Institute of the Beijing Centre for Diseases Prevention and Control (CDC) in China. Clinical data were reported by clinicians in four designated HIV treatment hospitals in Beijing: Peking Union Medical College Hospital, Beijing You'an Hospital, Beijing Ditan Hospital and 302 Military Hospital of China. Our analysis included all treatment‐naive PLWH who initiated therapy at one of these hospitals between 1 January 2010 and 31 December 2020 [11]. The data were taken directly from the hospital's records on the date that ART was prescribed.

PLWH aged 18–80 years who initiated ART in Beijing, China, were included in this study. Two age groups were identified: PLWH aged 18–49 years and those aged ≥50 years. Confidentiality was protected for individual information processing. The study protocol was approved by the Research Ethics Committee of the Beijing CDC. The data were based on clinical records that had already been de‐identified, so informed consent was unnecessary.

### 2.2 Definition of variables

Marital status was defined as single, married or living with a partner, divorced or separated, or widowed. Route of transmission included sexual intercourse (homosexual or heterosexual), people who inject drugs (PWID) or other. Overweight or obesity was defined as body mass index (BMI) ≥24 kg/m^2^, following the Working Group on Obesity in China [12, 13]. Laboratory testing was conducted at the hospital when PLWH initiated ART, including CD4 count, HIV viral load, hepatitis B virus (HBV) surface antigen (HBsAg), hepatitis C virus (HCV) antibody, total cholesterol (TC), triglyceride (TG), fasting blood glucose (FBG), aspartate aminotransferase (AST) and blood platelet count. Coinfection with HBV or HCV was defined as positive HBsAg or anti‐HCV. Hypercholesterolemia was defined as TC ≥5.2 mmol/l. Hypertriglyceridemia was defined as TG ≥1.7 mmol/l. Impaired fasting glucose (IFG) was defined as fasting blood glucose ≥6.1 mmol/l [14]. Estimated glomerular filtration rate (eGFR) was determined using the equation developed by the Chronic Kidney Disease Epidemiology Collaboration [15]. Renal insufficiency was defined as eGFR <90 ml/minute/1.73 m^2^ [15]. The AST to platelet ratio index (APRI) was calculated as the AST elevation (AST level divided by the upper limit of normal for the lab) and the platelet count per mm^3^ divided by 1000 [16]. Fibrosis was categorized as APRI ≥0.5 [17]. Comorbidities included overweight/obesity, hypercholesterolemia, hypertriglyceridemia, IFG, renal insufficiency and fibrosis. HIV‐related variables, such as AOI, and WHO HIV clinical staging were judged by clinicians. AOI was defined as having at least one AOI somewhere in the 3 months before ART initiation. Delayed ART initiation was defined as a lag time >30 days between the diagnosis and initiation of ART [18].

### 2.3 Statistical analysis

Continuous variables with normal distributions were reported as means (standard deviations), and differences between groups were compared using the *t*‐test; continuous variables with skewed distributions were reported as medians (interquartile ranges) and compared using the Mann−Whitney *U* test, and all categorical variables were presented as numbers (percentages) and compared using the chi‐square test. We conducted multivariate backward stepwise logistic regression to analyse the associations between each factor and PLWH having one or more comorbidities. We incorporated factors based on prior research and available data, including the following candidate variables: age (<50 or ≥50 years), gender (female or male), marital status (single, married or cohabitating, divorced or separated, widowed), route of transmission (homosexual, heterosexual, PWID, other), WHO Stage (Stage I, II, III or IV), CD4 counts (<200 or ≥200 cells/μl), plasma HIV RNA load (≤100 or >100 copies/ml), time intervals from HIV diagnosis to ART initiation (≤30 days, 31–90 days, 91–180 days, >180 days), HBV/HCV coinfection (yes or no) and AOI (yes or no). Detailed information on missing values for each variable and strategies for managing them are presented in Table S1.

Temporal trends in the proportions of older PLWH were estimated using joinpoint regression. Comparability tests were performed on temporal trends in the proportion of males and of infection by sexual intercourse (homosexual or heterosexual), CD4 counts <200 cells/μl and delayed initiation of ART among PLWH aged <50 and ≥50 years. Joinpoint regression analysis was used to identify years with changes in the linear slope of the temporal trend. Best‐fitting points were chosen at significant changes in proportion. This approach had two major advantages: it identified change points and estimated the magnitudes of increases or decreases over each interval by estimating the annual percent change (APC). All statistical analyses were performed using SPSS 23.0 (IBM Corp., Armonk, NY, USA) and the Joinpoint Regression Program (version 5.0.2., 2023; Information Management Services, Inc., Calverton, MD, USA) [19]. In all analyses, *p* values lower than 0.05 were considered statistically significant.

## 3 RESULTS

**Figure 1:**
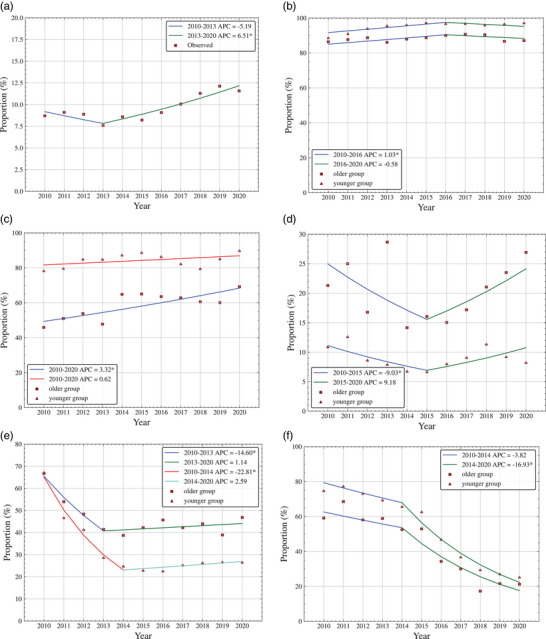
(a) Temporal trend in the proportion of treatment‐naïve PLWH aged ≥50 years in Beijing, China, 2010–2020. Final selected model: 1 joinpoint. (b) Temporal trend in the proportion of male treatment‐naïve PLWH by age group in Beijing, China, 2010–2020. Final selected model: older group, 1 joinpoint; younger group, 1 joinpoint; failed to reject parallelism. (c) Temporal trend in the proportion of transmission through homosexual contact by age group in Beijing, China, 2010–2020. Final selected model: older group, 0 joinpoint; younger group, 0 joinpoint; rejected parallelism. (d) Temporal trend in the proportion of transmission through heterosexual contact by age group in Beijing, China, 2010–2020. Final selected model: older group, 1 joinpoint; younger group, 1 joinpoint; failed to reject parallelism. (e) Temporal trend in the proportions of treatment‐naïve PLWH with CD4 counts less than 200 cells/μl by age group in Beijing, China, 2010–2020. Final selected model: older group, 1 joinpoint; younger group, 1 joinpoint; rejected parallelism. (f) Temporal trend in the proportion of delayed ART initiation among treatment‐naïve PLWH by age group in Beijing, China, 2010–2020. Final selected model: older group, 1 joinpoint; younger group, 1 joinpoint; failed to reject parallelism. *Indicates that the APC is significantly different from zero at the alpha = 0.05 level. Abbreviations: APC, annual percent change; ART, antiretroviral therapy; PLWH, people living with HIV.

In all, 23,622 PLWH who initiated ART between 2010 and 2020 were included in this study, of whom 2261 (9.57%) were aged ≥50 years, and 21,361 (90.43%) were aged <50 years. Most (72.27%) older PLWH were aged 50–59 years, 20.21% were aged 60–69 years and 7.52% were aged 70–80 years. As shown in Figure 1A, joinpoint regression modelling identified an inflection point in 2013 in the annual proportion of PLWH aged ≥50 years. The initial trend showed no evidence of change from 2010 to 2013. The subsequent trend had a statistically significant increase from 7.58% in 2013 to 11.58% in 2020 (APC = 6.51%; 95% confidence interval [*CI*] 3.90%, 9.19%; *p* = 0.001). The demographic characteristics of the participants, stratified by age, are shown in Table 1. The study population was dominated by men, with a higher proportion of women in the older group. Annual trends in the proportion of male PLWH by age group are presented in Figure 1B. No differences in the two trends were found (*p* = 0.077), with a statistically significant increase over 2010–2016 (APC = 1.03%; 95% *CI* 0.39%, 1.68%; *p* = 0.004) and a fluctuation from 2016 to 2020. In both age groups, more were Beijing residents than were not. A much higher proportion of PLWH were married or cohabitating in the older group. As with younger PLWH, older PLWH were primarily infected through homosexual contact, followed by heterosexual contact, PWID and other (including unknown). Figure 1C shows annual trends by age group in the proportion of transmissions through homosexual contact. The trends increased without an inflection point and were significantly different (*p* = 0.008). However, the increase was only statistically significant in the older group (APC = 3.32%; 95% *CI* 1.34%, 5.33%; *p* = 0.004). Figure 1D presents annual trends in the proportion of PLWH transmission through heterosexual contact by age group. No differences were found in the two trends (*p* = 0.280), with a significantly decreasing trend observed from 2010 to 2015 (APC = −9.03%; 95% *CI* −16.85%, −0.46%; *p* = 0.041) and fluctuations observed from 2015 to 2020.

**Table 1:** Demographic characteristics comparison between treatment‐naïve PLWH by age group in Beijing, China, 2010–2020

Variables	<50 years (*N* = 21,361)	≥50 years (*N* = 2261)	*p* value
Age, Median (IQR), years	30 (26, 36)	55 (52, 60)	<0.001
Male, *n* (%)	20,534 (96.17)	2003 (88.59)	<0.001
Registered residence			<0.001
Beijing	4250 (19.90)	1050 (46.44)	
Outside Beijing	17,111 (80.10)	1211 (53.56)	
Marital status^a^, *n* (%)			<0.001
Single	15,982 (75.58)	207 (9.35)	
Married/cohabitating	4177 (19.75)	1623 (73.27)	
Divorced or separated	952 (4.50)	290 (13.09)	
Widowed	35 (0.17)	95 (4.29)	
Route of transmission, *n* (%)			<0.001
Homosexual	18,020 (84.36)	1360 (60.15)	
Heterosexual	1915 (8.96)	448 (19.81)	
PWID	156 (0.73)	9 (0.40)	
Others	1270 (5.95)	444 (19.64)	

Abbreviations: PLWH, people living with HIV; PWID, people who inject drugs.

^a^ Marital status, data of marital status were not available for 1.01% of patients.

The HIV‐related and non‐HIV‐related characteristics of the participants by age are shown in Table 2. At ART initiation, HIV‐related characteristics or symptoms were more severe in the older group than in the younger one. A higher proportion of WHO HIV Stage II to IV and particularly Stage IV, a higher proportion of AOI, a higher proportion of CD4 counts <200 cells/μl and higher levels of plasma HIV RNA load were seen in older PLWH than in younger ones. The older group also had a shorter median time from diagnosis to ART initiation.

**Table 2:** HIV‐related, non‐HIV‐related characteristics and treatment comparison between treatment‐naïve PLWH by age group in Beijing, China, 2010–2020

Variables	<50 years (*N* = 21,361)	≥50 years (*N* = 2261)	*p* value
WHO Stage, *n* (%)			<0.001
I	16,757 (78.45)	1593 (70.46)	
II	2324 (10.88)	271 (11.99)	
III	1104 (5.17)	160 (7.08)	
IV	1176 (5.51)	237 (10.48)	
CD4 counts^a^, *n* (%), cells/μl			<0.001
<200	5614 (27.41)	960 (43.72)	
200–349	7140 (34.85)	689 (31.38)	
350–499	4784 (23.35)	332 (15.12)	
≥500	2947 (14.39)	215 (9.79)	
Plasma HIV RNA load^b^, mean (SD), log_10_ copies/ml	4.43 (0.85)	4.63 (0.88)	<0.001
Time intervals from HIV diagnosis to ART initiation, Median (IQR), days	29 (13,113)	22 (13, 47)	<0.001
Delayed initiation of ART, *n* (%)	10,403 (48.70)	847 (37.46)	<0.001
HBV/HCV coinfection, *n* (%)	1195 (5.59)	135 (5.97)	0.285
AOI, *n* (%)	584 (2.73)	97 (4.29)	<0.001
ART regimen, *n* (%)			<0.001
EFV+3TC+TDF	16,308 (76.34)	1584 (70.06)	
EFV+3TC+AZT	2250 (10.53)	303 (13.40)	
LPV/r+3TC+TDF	606 (2.84)	89 (3.94)	
NVP+3TC+AZT	444 (2.08)	48 (2.12)	
TAF/FTC/EVG/c	328 (1.54)	32 (1.42)	
others	1425 (6.67)	205 (9.07)	
Numbers of comorbidities, *n* (%)			<0.001
0	11,701 (54.78)	662 (29.28)	
1	6543 (30.63)	769 (34.01)	
2	2367 (11.08)	558 (24.68)	
3+	750 (3.51)	272 (12.03)	
Multimorbidity^c^, *n* (%)	3117 (14.59)	830 (36.71)	<0.001

Abbreviations: AOI, AIDS‐related opportunistic illnesses; ART, antiretroviral therapy; AZT, zidovudine; EFV, efavirenz; EVG/c, elvitegravir‐cobicistat; FTC, emtricitabine; HBV, hepatitis B virus; HCV, hepatitis C virus; IQR, interquartile range; LPV/r, lopinavir/ritonavir; NVP, nevirapine; PLWH, people living with HIV; TAF, tenofovir alafenamide; 3TC, lamivudine; TDF, tenofovir; WHO, World Health Organization.

^a^ CD4 counts, data of CD4 counts were not available for 3.98% of patients.

^b^ Plasma HIV RNA load, data of plasma HIV RNA load were not available for 24.48% of patients.

^c^ Multimorbidity, the presence of two or more comorbidities.

Temporal trends by age group in the proportions of CD4 counts <200 cells/μl in treatment‐naïve PLWH are shown in Figure 1E. The two groups showed significantly different trends (*p* = 0.002), with inflection points in 2013 for the older and 2014 for the younger groups. Both groups experienced a sharp decrease in the initial trend (APC = −14.6%; 95% *CI* −24.31%, −3.64%; *p* = 0.019 vs. APC = −22.81%; 95% *CI* −27.17%, −18.18%; *p* < 0.001), followed by a stable period. Temporal trends in the rate of delayed ART initiation among PLWH by age group are shown in Figure 1F. No differences in the two trends were found (*p* = 0.463), and the initiation delay improved for both groups over the decade, especially from 2014 to 2020 (APC = −16.93%; 95% *CI* −20.97%, −12.69%; *p* < 0.001). Nevertheless, a difference remained between the two groups, and the proportion of delayed ART initiation was 10% lower in the older group. All the aforementioned trends among treatment‐naïve PLWH in Beijing, China from 2010 to 2020, as determined by joinpoint analysis, are presented in Table S2.

As the timeframe of this study includes year of 2020, when the COVID‐19 epidemic existed, which might  influence the diagnosis and treatment of HIV acquisition due to lockdowns and reduced access to clinical care, a sensitivity analysis was conducted that excluded data from 2020, presenting in Table S3, and Figure 1A—F. There were no changes in trends related to the proportion of transmission through heterosexual contact and less than 200 CD4 cells/μl. However, there were noticeable shifts at inflection points in the trends for older PLWH, male PLWH, transmission through homosexual contact and delayed ART initiation.

**Figure 2:**
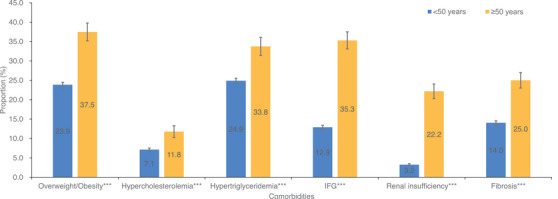
Non‐HIV‐related risk factors for treatment‐naïve PLWH by age group in Beijing, China, 2010–2020. ^***^Indicates *p* < 0.001. Overweight or obesity, BMI data were not available for 23.96% of patients. Hypertriglyceridemia and TG data were not available for 25.43% of patients. Hypercholesterolemia, data of TC were not available for 21.26% of patients. Impaired fasting glucose and FBG data were not available for 19.28% of patients. Renal insufficiency and eGFR data were not available for 16.86% of patients. Fibrosis and APRI data were not available for 18.06% of patients. Abbreviations: IFG, impaired fasting glucose; PLWH, people living with HIV.

A significantly higher prevalence of comorbidities was seen in the older group (Figure 2). Furthermore, older PLWH were more likely to have two or more comorbidities (Table 2). We assessed factors that could be associated with the presence of an HIV comorbidity (see Table 3). PLWH in the older group were more than twice as likely to have a comorbidity than those in the younger group. Other risk factors included male, non‐single, transmission through heterosexual contact or PWID, WHO Stage IV, coinfection with HBV/HCV and a threshold of 200 CD4 cells/μl at ART initiation.

**Table 3:** Factors associated with having HIV comorbidity (Beijing, 2010–2020)

Variables	OR	95% *CI* for OR		*p*
		Lower	Upper	
Age group, years				
<50	References	—	—	—
≥50	2.120	1.900	2.364	<0.001
Gender				
Female	References	—	—	—
Male	1.213	1.042	1.411	0.013
Marital status				<0.001
Single	References	—	—	—
Married/cohabitating	1.725	1.607	1.851	<0.001
Divorced or separated	1.940	1.713	2.198	<0.001
Widowed	1.817	1.226	2.692	0.003
Route of transmission				0.001
Homosexual	References	—	—	—
Heterosexual	1.175	1.058	1.305	0.003
PWID	1.647	1.180	2.297	0.003
Others	1.000	0.889	1.125	0.999
WHO Stage				0.029
I	References	—	—	—
II	0.984	0.901	1.074	0.714
III	0.933	0.825	1.056	0.271
IV	1.176	1.040	1.331	0.010
Plasma HIV RNA load, copies/ml				0.003
<100	References	—	—	—
≥100	0.859	0.689	1.069	0.173
Unknown (missing)	0.774	0.618	0.970	0.026
HBV/HCV coinfection				
No	References	—	—	—
Yes	1.476	1.313	1.659	<0.001
CD4 counts, cells/μl				
≥200	References	—	—	—
<200	1.088	1.021	1.160	0.009

Abbreviations: *CI*, confidence interval; HBV, hepatitis B virus; HCV, hepatitis C virus; PWID, people who inject drugs; WHO, World Health Organization.

**Figure 3:**
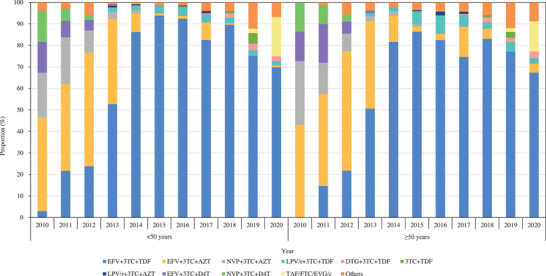
Temporal trend of ART selection among treatment‐naïve PLWH by age group in Beijing, China, 2010–2020. Abbreviations: AZT, zidovudine; EFV, efavirenz; EVG/c, elvitegravir‐cobicistat; FTC, emtricitabine; LPV/r, lopinavir/ritonavir; NVP, nevirapine; TAF, tenofovir alafenamide; 3TC, lamivudine; TDF, tenofovir.

No significant differences were seen in ART selection between older and younger PLWH. Between 2010 and 2020, efavirenz, lamivudine and zidovudine (EFV+3TC+AZT) were the most commonly used ART regimens, accounting for approximately 50%. This shifted to EFV+3TC+tenofovir (TDF) from 2013 to 2020; the proportions administered fluctuated but were over 50% at all times (Table 2). The temporal trend of ART selection among PLWH by age group is shown in Figure 3.

## 4 DISCUSSION

Our study showed that, among treatment‐naïve PLWH, the proportion of older individuals was stable and low before 2013 but thereafter showed a slight upward trend. This may be due to both the growing number of newly reported older PLWH and changes in the national ART strategy. The eligibility criteria for ART initiation were adjusted from less than 200–350 CD4 cells/μl in 2008 to 500 cells/μl in 2014, and finally, no CD4 cell count threshold in 2016 [20]. There was a large proportion of less than 500 CD4 cells/μl in older PLWH. Therefore, the proportion of older PLWH initiating ART began an upward trend in 2014 and is expected to further increase in the future.

We observed a fluctuating upward trend in the proportion of male treatment‐naïve PLWH, and this trend did not significantly differ between the older and younger groups. This may be due to the increasing proportion of PLWH transmitted by homosexual contact in recent years, particularly among the older group, which demonstrated a significant increase over the decade; alternatively or additionally, the trend may reflect the impact of the government's expansion of healthcare availability. The Chinese CDC has had a social app since 2013, adding HIV testing sites in Beijing serving approximately 700 men who have sex with men (MSM) per month in 2017 [21]. HIV cases in China among MSM have increased rapidly in recent years, with most localized in large Chinese cities [22]. Beijing, Guangdong, Sichuan, Jiangsu and Liaoning have among China's highest prevalence of PLWH with MSM transmission [23]. This increasing trend could be attributable to the gradual expansion in the disclosure of homosexual behaviour in China in recent years. The growing use of social media has facilitated social support catering to MSM, including gay dating apps. This has contributed to a greater likelihood that individuals will disclose their sexual orientation to various parties, including healthcare professionals [24]. Approximately 60% of MSM in China report disclosing their sexual orientation to someone, with more than 15% disclosing it to healthcare professionals [25]. Notably, the odds of disclosure to a healthcare professional are higher among MSM having had HIV testing or self‐reporting as living with HIV [25].

In our study, although the rate of MSM was lower in the older group than the younger group, it remained the most common route of HIV in this group and was much higher than that found in a cross‐sectional study in Taiwan (49.0%) [26] and a cohort study in Latin America (24%) [27]. However, the populations in those two studied were PLWH who had follow‐up HIV care at a hospital, which may account for the difference. It is crucial to tailor interventions to older MSM to prevent transmission in large cities. In addition, a higher proportion of HIV cases were classified as unknown transmission in the older group. This pattern could result from underlying factors, including low perception of and misconceptions regarding transmission risks, stigma and subsequent underreporting of transmission from MSM, along with poor risk assessment and sexual history collection by healthcare providers [28].

Late initiation of ART was defined as having CD4 counts <200 cells/μl or having a clinical AIDS diagnosis at ART initiation [29]. The proportion with CD4 counts <200 cells/μl significantly decreased from 2010 to 2013 among PLWH in older and younger groups, and delayed initiation of ART significantly improved from 2014 to 2020 for both groups. This may be due to the changing CD4 thresholds for ART initiation in Chinese guidelines. In 2002, ART initiation was indicated for CD4 counts <200 cells/μl. This threshold increased to 350 cells/μl in 2008 and 500 cells/μl in 2014 [18].

Nevertheless, older PLWH had a significantly higher proportion of CD4 counts <200 cells/μl, and experienced a slower decrease than the younger group. In addition, the proportion of CD4 counts <200 cells/μl in older PLWH is substantially higher than that reported in a report covering 31 European countries, where the corresponding proportion was 27.4% [28]. Although older PLWH initiated ART earlier, their HIV‐related characteristics or symptoms were severe than those of younger adults, which reflects the late initiation of ART among older PLWH. Late ART initiation is a significant impediment to preventing and effectively treating HIV. Early diagnosis and treatment in older individuals is particularly challenging because early signs and symptoms can be attributed to diseases of ageing, and neither these individuals nor their care providers perceive them to be at risk for HIV [30]. A cross‐sectional nationwide online survey conducted in China likewise found that MSM aged ≥50 years had limited HIV‐related knowledge and tended not to have been tested for HIV [31]. Future efforts should focus on provider‐initiated HIV testing and counselling specifically targeted at older PLWH and MSM, particularly in high‐prevalence areas.

This study found that older PLWH were more than twice as likely to have one or more comorbidities than younger PLWH at the initiation of ART, similar to older PLWH with access to ART in many Western countries [32]. It is also widely acknowledged that PWID is a major driver for cardiovascular diseases (CVDs), which have historically been more prevalent among PLWH [33]. This study identified it as a risk factor for comorbidities. The increased risk for comorbidities in male PLWH is opposed to that of a study conducted in South Africa [34]. This inconsistency may be attributable to the large proportion of male PLWH in this study or other differences between the populations.

HIV acquisition and the associated immune activation are independent risk factors for many comorbidities [35]. Our multivariate analysis confirmed that higher‐severity HIV status, including WHO Stage IV, HBV/HCV coinfection and CD4 counts <200 cells/μl, was associated with a higher risk for comorbidities. It has been found that PLWH, particularly older PLWH, have a high burden of comorbidities relative to HIV‐negative populations [36, 37]. In addition, lifelong use of ART exerts systemic influences on comorbidities [35]. However, no statistically significant differences were seen between older and younger PLWH on ART regimens in this study. There were multiple potential toxicities of ART to consider when selecting a regimen for older PLWH, including bone and renal effects related to TDF, weight gain related to integrase strand transfer inhibitors and tenofovir alafenamide (TAF), neurocognitive or neuropsychiatric toxicities and hyperlipidaemia related to EFV [38].

The intersection of ageing‐related and HIV‐related social services and support systems should be enhanced. We strongly recommend that clinicians pay greater attention to comorbidities in older PLWH at the initiation of ART to better prevent and manage further comorbidities. Furthermore, it is necessary for health systems to reconsider care strategies and meet the growing needs of older PLWH. For example, improved screening algorithms are needed to detect metabolic disease in older PLWH. At present, there are no validated CVD risk assessment tools specifically designed for PLWH. The most prominent ones developed hitherto for the general population include the US‐based pooled cohort equations, the Framingham risk functions and the Europe‐based systematic coronary risk evaluation [39]. However, these models tend to underestimate the actual CVD risk in PLWH. There is also an HIV‐specific CVD prediction model called Data Collection on Adverse Events of Anti‐HIV Drugs. Nevertheless, its performance is modest, particularly when applied to cohorts in the United States [39]. In addition, it is increasingly important for HIV physicians to monitor drug–drug interactions, as they consider the choices of ART regimens [40].

Our study had several strengths. First, the data were routinely collected through a standard hospital‐based data‐collection platform, ensuring their validity and reliability. In addition, all subjects were ART‐naïve PLWH; thus, we excluded any adverse effects caused by ART regimens to focus on the effects of ageing and HIV status. However, our study also had notable limitations. First, only already available clinical or demographic parameters were analysed, and some variables had a missing rate of more than 10%, causing us to create a separated “missing” group in the regression model. Second, some comorbidities were not systematically examined, including hypertension, osteoporosis and malignancy, as the information examined was mainly from laboratory data, accumulated during clinical care. However, laboratory data are more reliable than measurements. Finally, the healthy lifestyle factors and socio‐economic status were not included. Future studies should pay closer attention to the more detailed characteristics of PLWH.

## 5 CONCLUSIONS

Our study highlights a significant increase in the proportion of PLWH aged ≥50 years and the important role of MSM in Beijing's HIV epidemic among older PLWH. In addition, widespread testing and earlier diagnosis should be implemented to address the late initiation of ART among older PLWH. Furthermore, the significant proportion of older PLWH in Beijing with multimorbidities indicates that HIV physicians should provide high‐quality care for screening, preventing and treating age‐related diseases, ideally through a multidisciplinary approach. Finally, under universal health coverage, older PLWH have lifelong access to comprehensive services, prioritizing ending HIV transmission through a strong monitoring system, integrated care and specialist access. This approach provides holistic care, ensuring accessibility and sustainability for older PLWH.

## COMPETING INTERESTS

DW, MZ, PW, JZ, YM, JL and FC disclose no competing interests.

## AUTHORSʼ CONTRIBUTIONS

The authors’ responsibilities were as follows: DW wrote the manuscript. MZ contributed to conceptualization, writing, review, and editing. PW, JZ and YM contributed to the statistical analysis, discussion and reviewed/edited the manuscript. MZ, JL and FC were involved in the study design and data collection, contributed to the discussion, and reviewed and critically revised the manuscript.

## FUNDING

This work was supported by the National Key R&D Program of China during the 14th Five‐Year Plan Period [Project No. 2021ZD0114102], the National Natural Science Foundation of China [Project No. 71874100], Beijing Municipal Science & Technology Commission [Project No. D171100006717002], Incubation Fund of Vanke School of Public Health at Tsinghua University [Project No. 2021PY002] and Sanming Project of Medicine in Shenzhen [No. SZSM202111001].

## Supporting information


**Table S1**: Missing rates of each variable and management of missing data.
**Table S2**: Trends in the proportion of older, male, homosexual, heterosexual, CD4 counts <200 cells/µL, and delayed ART initiation among treatment‐naïve PLWH in Beijing, China from 2010 to 2020, as determined by Joinpoint analysis.
**Table S3**: Trends in the proportion of older, male, homosexual, heterosexual, CD4 counts <200 cells/µL, and delayed ART initiation among treatment‐naïve PLWH in Beijing, China from 2010 to 2019, as determined by Joinpoint analysis.
**Figure S1A**: Temporal trend in the proportion of treatment‐naïve PLWH aged ≥50 years in Beijing, China, 2010–2019.
**Figure S1B**: Temporal trend in the proportion of male treatment‐naïve PLWH by age group in Beijing, China, 2010–2019.
**Figure S1C**: Temporal trend in the proportion of transmission through homosexual contact by age group in Beijing, China, 2010–2019.
**Figure S1D**: Temporal trend in the proportion of transmission through heterosexual contact by age group in Beijing, China, 2010–2019.
**Figure S1E**: Temporal trend in the proportions of treatment‐naïve PLWH with CD4 counts less than 200 cells/µL by age group in Beijing, China, 2010–2019.
**Figure S1F**: Temporal trend in the proportion of delayed ART initiation among treatment‐naïve PLWH by age group in Beijing, China, 2010–2019.

## Data Availability

Research data are not shared.
