# Supplementary material for: Treatment‐naïve people living with HIV aged 50 years or older in Beijing, China, 2010–2020: joinpoint regression model analysis of surveillance data
Source: J Int AIDS Soc. 2023 Dec 6;26(12):e26193. doi: 10.1002/jia2.26193 (PMC10698805; doi:10.1002/jia2.26193)
Supplement: Supplementary file 1 — Table S1: Missing rates of each variable and management of missing data. Table S2: Trends in the proportion of older, male, homosexual, heterosexual, CD4 counts <200 cells/µL, and delayed ART initiation among treatment‐naïve PLWH in Beijing, China from 2010 to 2020, as determined by Joinpoint analysis. Table S3: Trends in the proportion of older, male, homosexual, heterosexual, CD4 counts <200 cells/µL, and delayed ART initiation among treatment‐naïve PLWH in Beijing, China from 2010 to 2019, as determined by Joinpoint analysis. Figure S1A: Temporal trend in the proportion of treatment‐naïve PLWH aged ≥50 years in Beijing, China, 2010–2019. Figure S1B: Temporal trend in the proportion of male treatment‐naïve PLWH by age group in Beijing, China, 2010–2019. Figure S1C: Temporal trend in the proportion of transmission through homosexual contact by age group in Beijing, China, 2010–2019. Figure S1D: Temporal trend in the proportion of transmission through heterosexual contact by age group in Beijing, China, 2010–2019. Figure S1E: Temporal trend in the proportions of treatment‐naïve PLWH with CD4 counts less than 200 cells/µL by age group in Beijing, China, 2010–2019. Figure S1F: Temporal trend in the proportion of delayed ART initiation among treatment‐naïve PLWH by age group in Beijing, China, 2010–2019. [file JIA2-26-e26193-s001.docx]

**Supplemental Table 1.** Missing rates of each variable and management of missing data.

| Variables | Missing, N (%) | | | Management of missing data |
| --- | --- | --- | --- | --- |
|  | Total  (N = 23,622) | ≥50 years  (N = 2,261) | <50 years  (N = 21,361) |  |
| Fasting blood glucose | 4,555 (19.28) | 457 (20.21) | 4,098 (19.18) | Complete case analysis was conducted in univariable analysis (exclude cases analysis by analysis).  Missing value were excluded in multivariable regression model (n = 1,198, 5.07%). |
| Total cholesterol | 5,023 (21.26) | 555 (24.55) | 4,468 (20.92) |  |
| Triglyceride | 6,007 (25.43) | 670 (29.63) | 5,337 (24.98) |  |
| Serum creatinine | 3,983 (16.86) | 408 (18.05) | 3,575 (16.74) |  |
| Blood platelet count | 3,656 (15.48) | 377 (16.67) | 3,279 (15.35) |  |
| Aspartate aminotransferase | 3,585 (15.18) | 383 (16.94) | 3,202 (14.99) |  |
| Weight | 5,166 (21.87) | 507 (22.42) | 4,659 (21.81) |  |
| Height | 5,591 (23.67) | 544 (24.06) | 5,047 (23.63) |  |
| CD4 counts | 941 (3.98) | 65 (2.87) | 876 (4.10) |  |
| Marital status | 261 (1.10) | 46 (2.03) | 215 (1.01) |  |
| Plasma HIV RNA load | 5,515 (23.35) | 465 (20.57) | 5,050 (23.64) | Complete case analysis was conducted in univariable analysis.  A “missing” group was created in multivariable regression model. |

**Supplemental Table 2.** Trends in the proportion of older, male, homosexual, heterosexual, CD4 counts <200 cells/μL, and delayed ART initiation among treatment-naïve PLWH in Beijing, China from **2010 to 2020**, as determined by Joinpoint analysis.

| **Variables** | **Period** | **APC (95%*CI*)** | ***P*** |
| --- | --- | --- | --- |
| **PLWH ≥50 years** |  |  |  |
| Trend 1 | 2010–2013 | -5.19 (-13.60, 4.05) | 0.211 |
| Trend 2 | 2013–2020 | 6.51 (3.90, 9.19) | **0.001** |
| AAPC | 2010–2020 | 2.86 (0.19, 5.60) | **0.036** |
| **Male PLWH** |  |  |  |
| Trend 1 | 2010–2016 | 1.03 (0.39, 1.68) | **0.004** |
| Trend 2 | 2016–2020 | -0.58 (-1.77, 0.62) | 0.315 |
| AAPC | 2010–2020 | 0.38 (-0.18, 0.95) | 0.185 |
| Test for parallelism | 2010–2020 | — | 0.077 |
| **Homosexual** |  |  |  |
| AAPC (elderly group) | 2010–2020 | 3.32 (1.34, 5.33) | **0.004** |
| AAPC (younger group) | 2010–2020 | 0.62 (-0.32, 1.57) | 0.168 |
| Test for parallelism | 2010–2020 | — | **0.008** |
| **Heterosexual** |  |  |  |
| Trend 1 | 2010–2015 | -9.03 (-16.85, -0.46) | **0.041** |
| Trend 2 | 2015–2020 | 9.18 (-0.21, 19.46) | 0.055 |
| AAPC | 2010–2020 | -0.34 (-6, 5.67) | 0.910 |
| Test for parallelism | 2010–2020 | — | 0.280 |
| **CD4 counts <200 cells/μL** |  |  |  |
| Elderly group |  |  |  |
| Trend 1 | 2010–2013 | -14.6 (-24.31, -3.64) | **0.019** |
| Trend 2 | 2013–2020 | 1.14 (-2.07, 4.45) | 0.424 |
| AAPC | 2010–2020 | -3.86 (-7.1, -0.52) | **0.024** |
| Younger group |  |  |  |
| Trend 1 | 2010–2014 | -22.81 (-27.17, -18.18) | **< 0.001** |
| Trend 2 | 2014–2020 | 2.59 (-0.55, 5.83) | 0.090 |
| AAPC | 2010–2020 | -8.44 (-10.6, -6.23) | **< 0.001** |
| Test for parallelism | 2010–2020 | — | **0.002** |
| **Delayed ART initiation** |  |  |  |
| Trend 1 | 2010–2014 | -3.82 (-12.37, 5.57) | 0.387 |
| Trend 2 | 2014–2020 | -16.93 (-20.97, -12.69) | **< 0.001** |
| AAPC | 2010–2020 | -11.92 (-15.7, -7.96) | **< 0.001** |
| Test for parallelism | 2010–2020 | — | 0.463 |

Abbreviations: PLWH, people living with HIV; ART, antiretroviral therapy; APC, annual percent change; AAPC, average annual percent change; *CI*, confidence interval.

**Supplemental Table 3.** Trends in the proportion of older, male, homosexual, heterosexual, CD4 counts<200 cells/μL, and delayed ART initiation among treatment-naïve PLWH in Beijing, China from **2010 to 2019**, as determined by Joinpoint analysis.

| **Variables** | **Period** | **APC (95%*CI*)** | ***P*** |
| --- | --- | --- | --- |
| **PLWH ≥50 years** |  |  |  |
| Trend 1 | 2010–2015 | -1.64 (-6.09, 3.02) | 0.401 |
| Trend 2 | 2015–2019 | 10.8 (3.78, 18.3) | **0.010** |
| AAPC | 2010–2019 | 3.71 (0.68, 6.82) | **0.016** |
| **Male PLWH** |  |  |  |
| Trend 1 | 2010–2017 | 0.92 (0.42, 1.43) | **0.002** |
| Trend 2 | 2017–2019 | -1.45 (-5.09, 2.33) | 0.417 |
| AAPC | 2010–2019 | 0.39 (-0.45, 1.23) | 0.363 |
| Test for parallelism | 2010–2019 | — | 0.136 |
| **Homosexual** |  |  |  |
| Elderly group |  |  |  |
| Trend 1 | 2010–2015 | 7.02 (0.09, 14.44) | **0.048** |
| Trend 2 | 2015–2019 | -1.7 (-10.58, 8.07) | 0.662 |
| AAPC | 2010–2019 | 3.05 (-1.27, 7.57) | 0.169 |
| Younger group |  |  |  |
| Trend 1 | 2010–2014 | 2.97 (-0.8, 6.89) | 0.100 |
| Trend 2 | 2014–2019 | -1.46 (-4.03, 1.18) | 0.212 |
| AAPC | 2010–2019 | 0.49 (-1.2, 2.2) | 0.574 |
| Test for parallelism | 2010–2019 | — | **0.005** |
| **Heterosexual** |  |  |  |
| Trend 1 | 2010–2015 | -9.53 (-17.17, -1.18) | **0.029** |
| Trend 2 | 2015–2019 | 11.36 (-1.71, 26.16) | 0.085 |
| AAPC | 2010–2019 | -0.78 (-7.22, 6.12) | 0.820 |
| Test for parallelism | 2010–2019 | — | 0.299 |
| **CD4 counts <200 cells/μL** |  |  |  |
| Elderly group |  |  |  |
| Trend 1 | 2010–2013 | -14.07 (-24.1, -2.72) | **0.026** |
| Trend 2 | 2013–2019 | 0.34 (-3.78, 4.64) | 0.842 |
| AAPC | 2010–2019 | -4.71 (-8.27, -1.01) | **0.013** |
| Younger group |  |  |  |
| Trend 1 | 2010–2014 | -22.88 (-27.59, -17.87) | **< 0.001** |
| Trend 2 | 2014–2019 | 2.81 (-1.66, 7.49) | 0.170 |
| AAPC | 2010–2019 | -9.52 (-12.06, -6.91) | **< 0.001** |
| Test for parallelism | 2010–2019 | — | **0.003** |
| **Delayed initiation of ART** |  |  |  |
| Trend 1 | 2010–2015 | -5.27 (-10.09, -0.2) | **0.043** |
| Trend 2 | 2015–2019 | -21.49 (-27.07, -15.48) | **< 0.001** |
| AAPC | 2010–2019 | -12.86 (-16.25, -9.33) | **< 0.001** |
| Test for parallelism | 2010–2019 | — | 0.552 |

Abbreviations: PLWH, people living with HIV; ART, antiretroviral therapy; APC, annual percent change; AAPC, average annual percent change; *CI*, confidence interval.


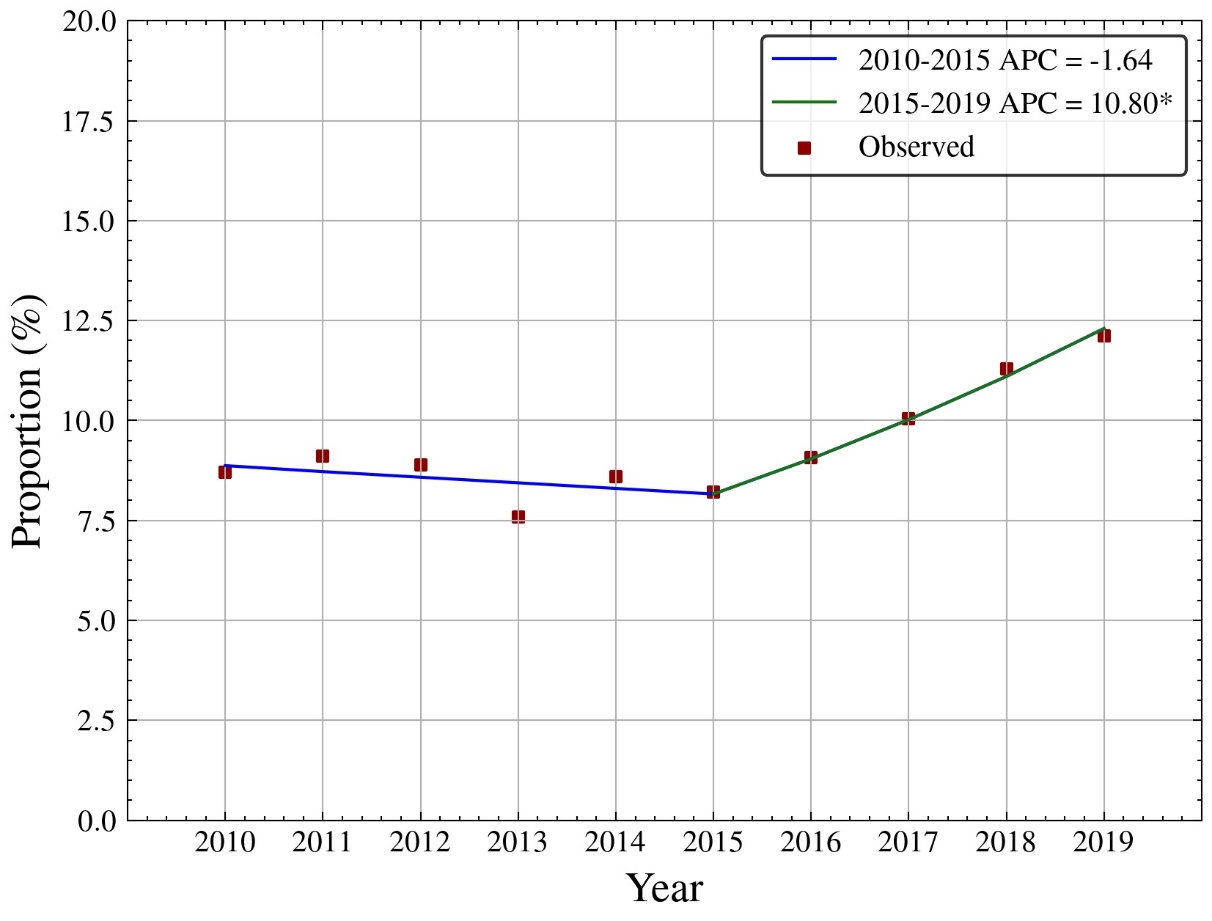


**Supplemental Figure 1A. Temporal trend in the proportion of treatment-naïve PLWH aged ≥50 years in Beijing, China, 2010–2019.**

Abbreviations: PLWH, people living with HIV; APC, Annual Percent Change.

^*^ Indicates that the APC is significantly different from Zero at the alpha = 0.05 level.

Final selected model: 1 Joinpoint.

**
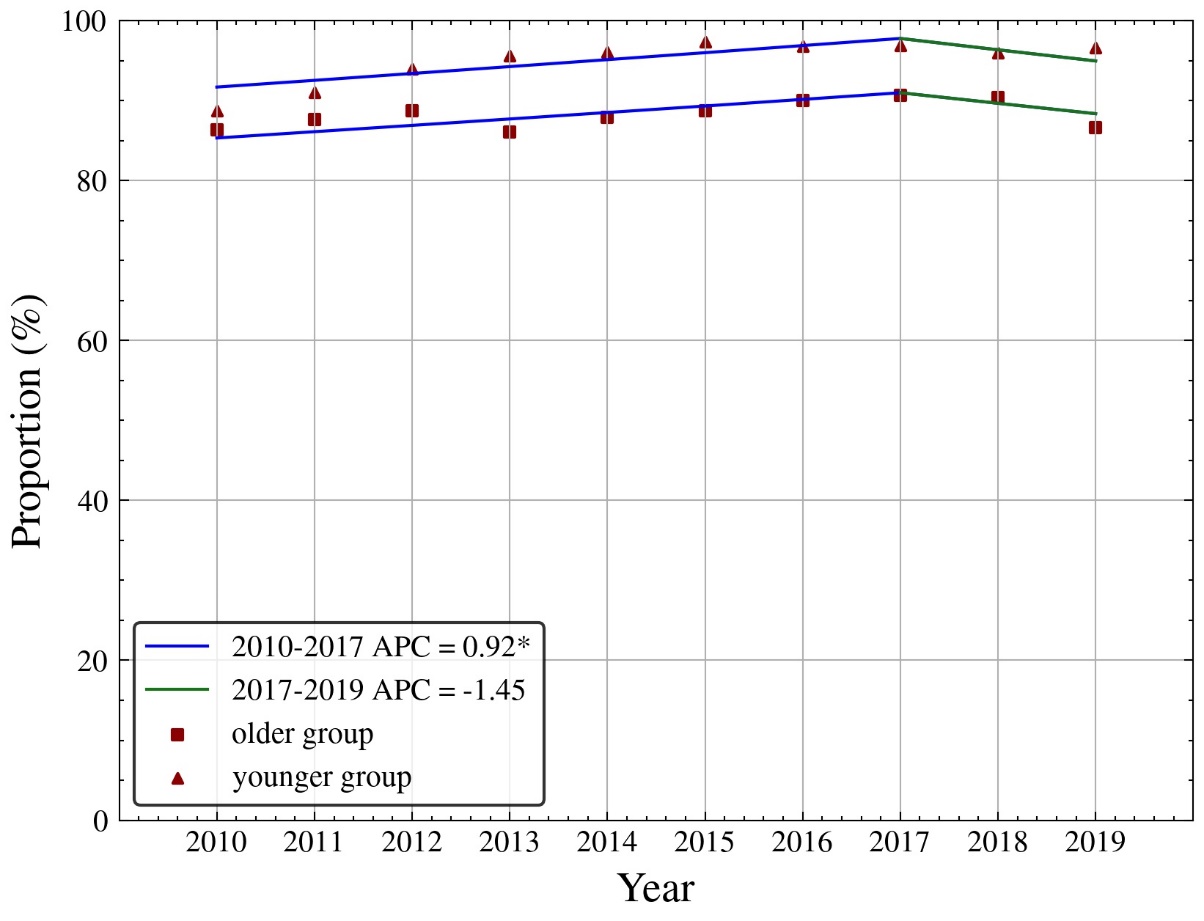
**

**Supplemental Figure 1B. Temporal trend in the proportion of male treatment-naïve PLWH by age group in Beijing, China, 2010–2019.**

Abbreviations: PLWH, people living with HIV; APC, Annual Percent Change.

^*^ Indicates that the APC is significantly different from Zero at the alpha = 0.05 level.

Final selected model: older group,1 Joinpoint; younger group, 1 Joinpoint; failed to reject parallelism.

**
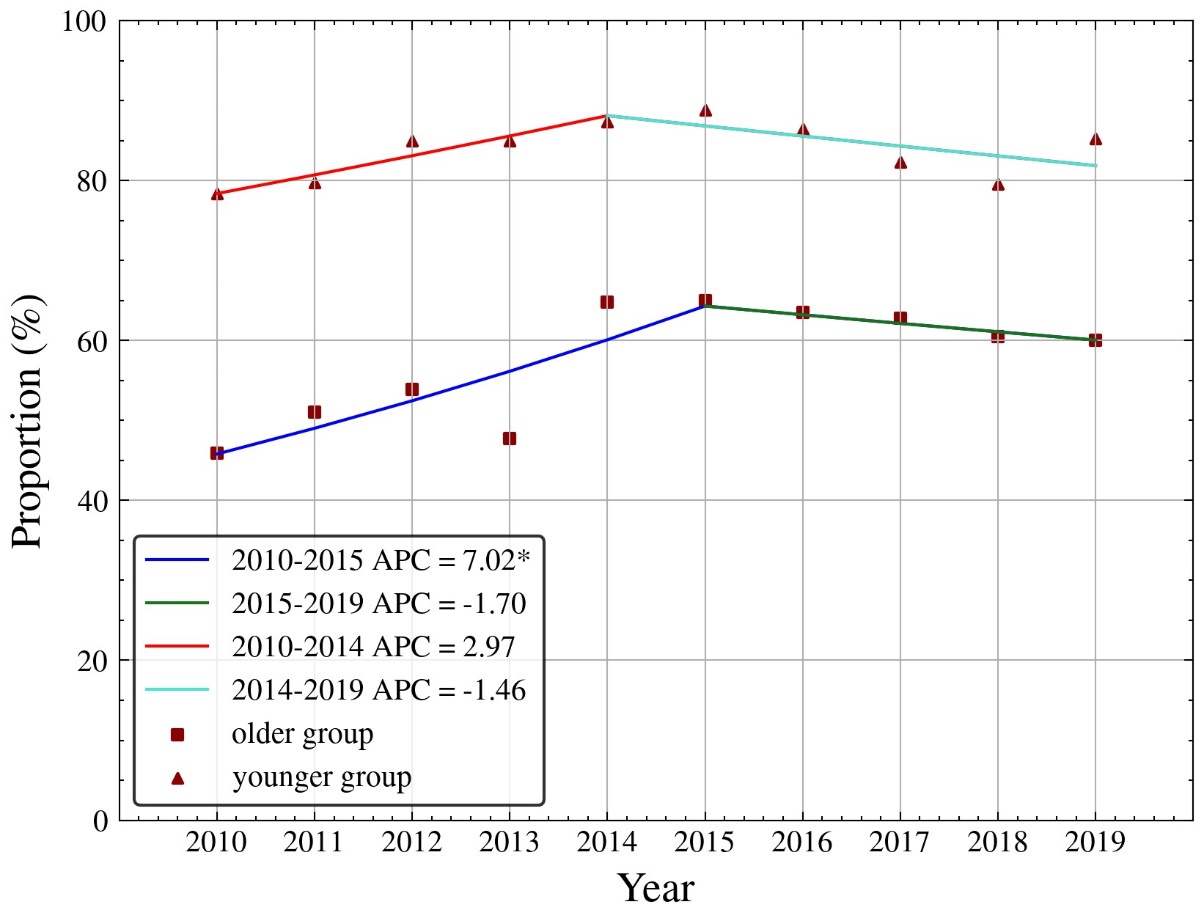
**

**Supplemental Figure 1C. Temporal trend in the proportion of transmission through homosexual contact by age group in Beijing, China, 2010–2019.**

Abbreviations: APC, Annual Percent Change.

^*^ Indicates that the APC is significantly different from Zero at the alpha = 0.05 level.

Final selected model: older group,1 Joinpoint; younger group, 1 Joinpoint; rejected parallelism.

**
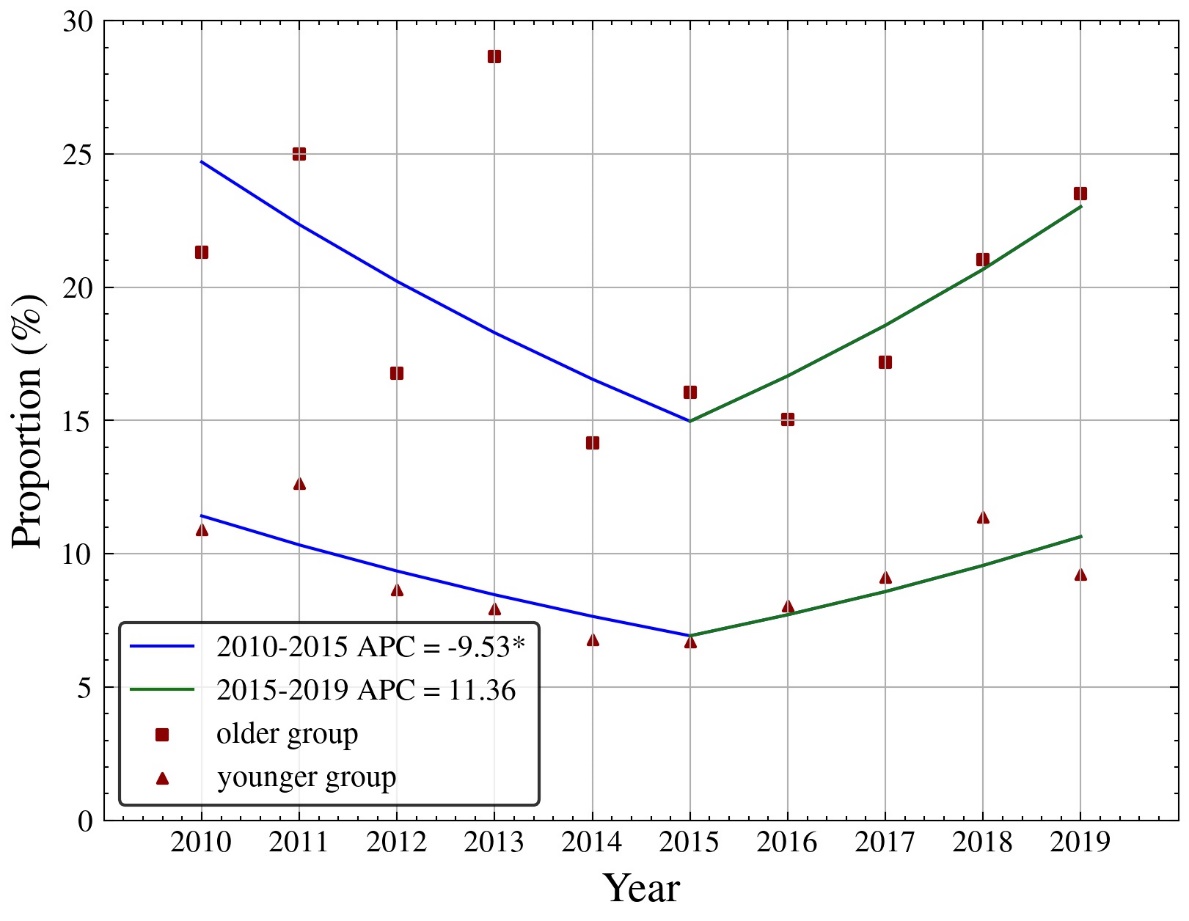
**

**Supplemental Figure 1D. Temporal trend in the proportion of transmission through heterosexual contact by age group in Beijing, China, 2010–2019.**

Abbreviations: APC, Annual Percent Change.

^*^ Indicates that the APC is significantly different from Zero at the alpha = 0.05 level.

Final selected model: older group,1 Joinpoint; younger group, 1 Joinpoint; failed to reject parallelism.

**
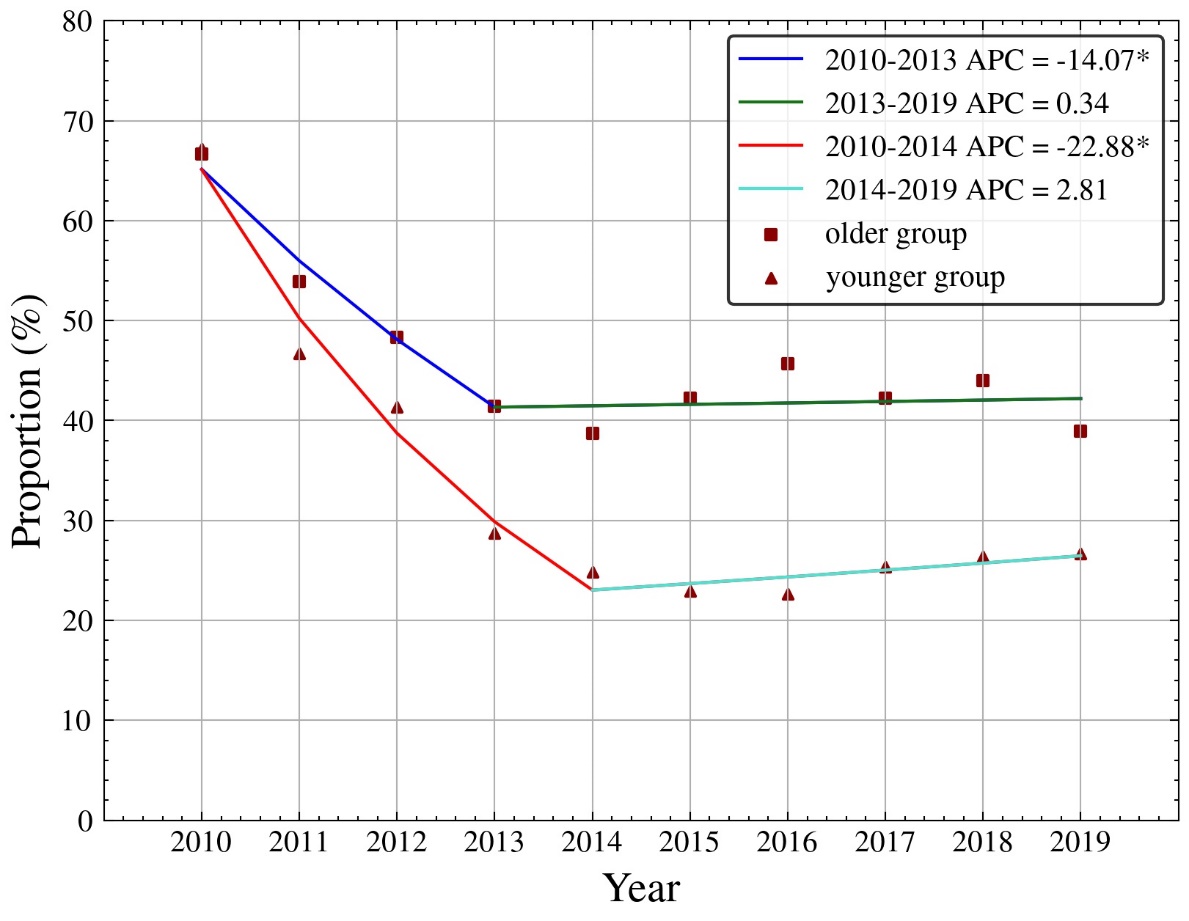
**

**Supplemental Figure 1E. Temporal trend in the proportions of treatment-naïve PLWH with CD4 counts less than 200 cells/μL by age group in Beijing, China, 2010–2019.**

Abbreviations: PLWH, people living with HIV; APC, Annual Percent Change.

^*^ Indicates that the APC is significantly different from Zero at the alpha = 0.05 level.

Final selected model: older group,1 Joinpoint; younger group, 1 Joinpoint; rejected parallelism.

**
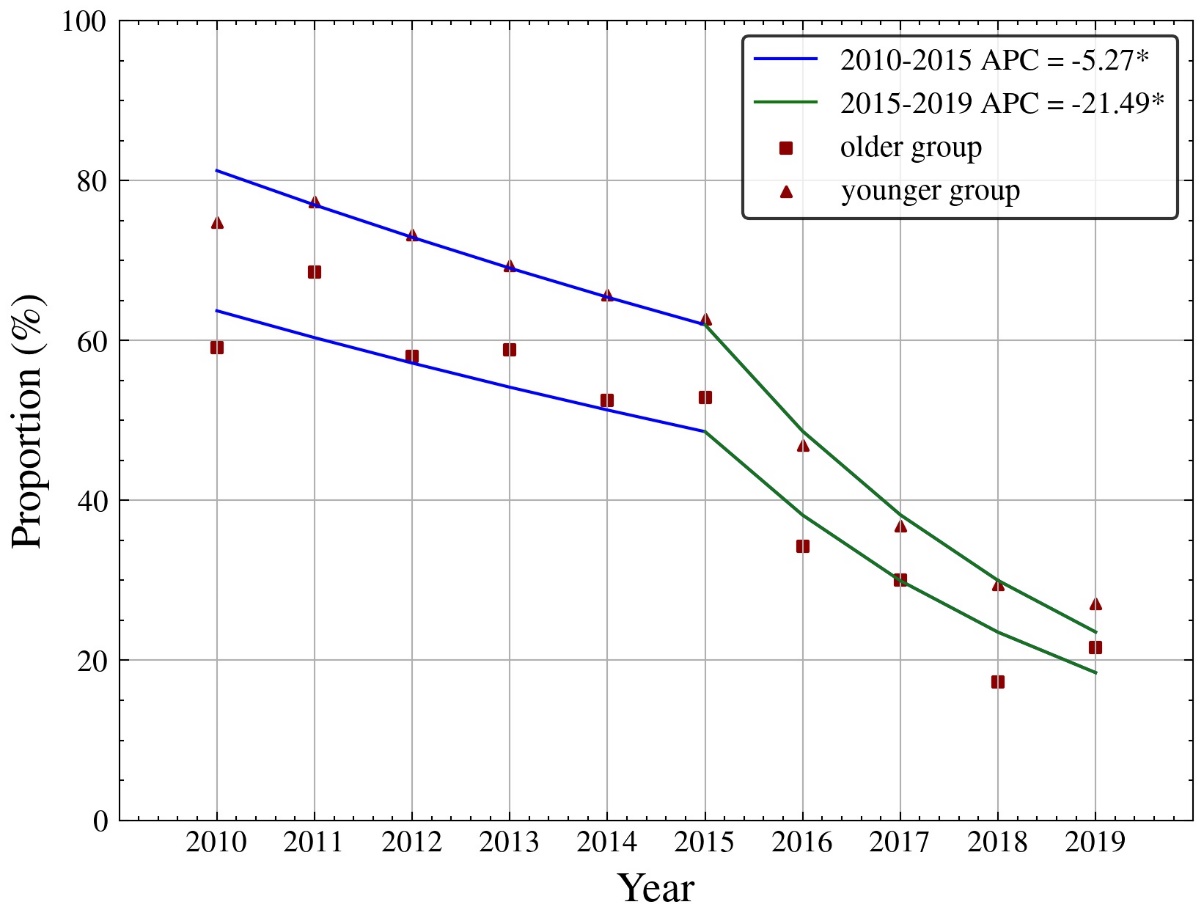
**

**Supplemental Figure 1F. Temporal trend in the proportion of delayed ART initiation among treatment-naïve PLWH by age group in Beijing, China, 2010–2019.**

Abbreviations: PLWH, people living with HIV; ART, antiretroviral therapy; APC, Annual Percent Change.

^*^ Indicates that the APC is significantly different from Zero at the alpha = 0.05 level.

Final selected model: older group,1 Joinpoint; younger group, 1 Joinpoint; failed to reject parallelism.
